# Supplementary material for: Novel Bile Salt Stabilized Vesicles-Mediated Effective Topical Delivery of Diclofenac Sodium: A New Therapeutic Approach for Pain and Inflammation
Source: Pharmaceuticals (Basel). 2022 Sep 5;15(9):1106. doi: 10.3390/ph15091106 (PMC9506322; doi:10.3390/ph15091106)
Supplement: Supplementary file 1 [file pharmaceuticals-15-01106-s001.zip › pharmaceuticals-1829351-supplementary.pdf]

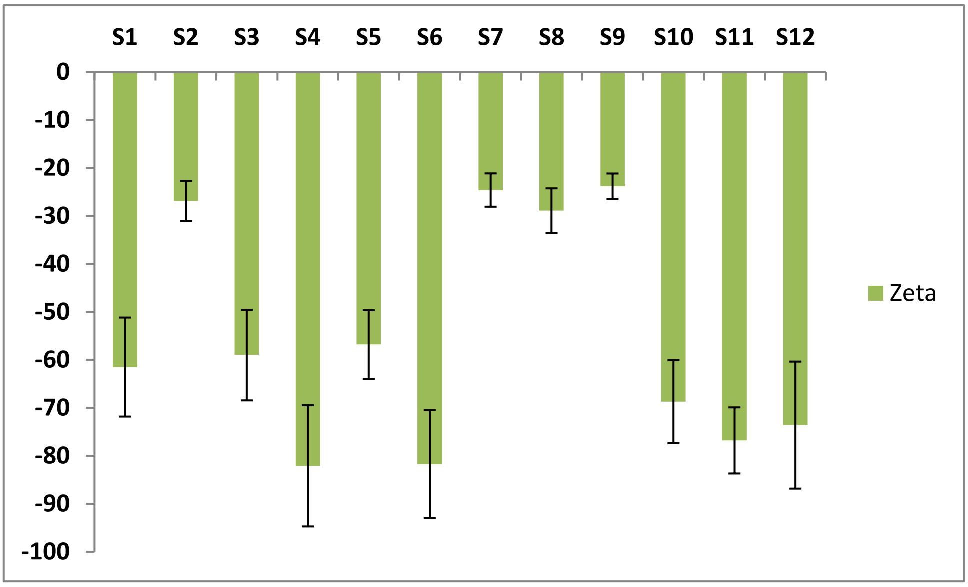

Figure S1. Zeta potential of DNa-BSVCs formulation.

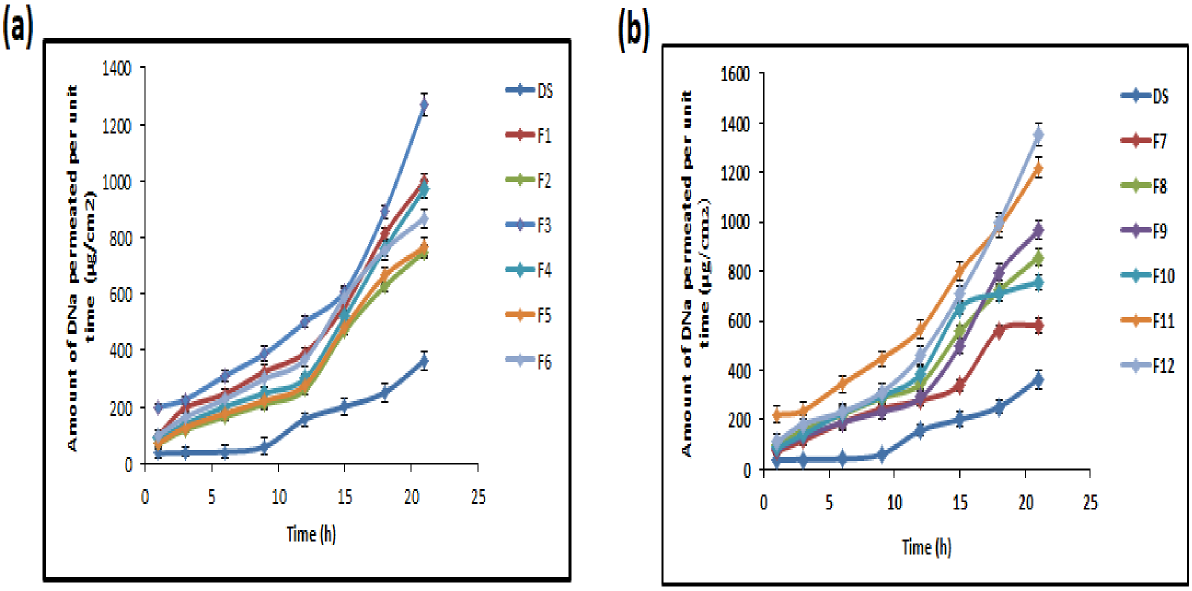

Figure S2. Permeation profile of DNa from drug solution and different BSVCs formulation: (**a**) F1–F6 and (**b**) F7–F12.
